# A density-based matrix transformation clustering method for electrical load

**DOI:** 10.1371/journal.pone.0272767

**Published:** 2022-08-11

**Authors:** Naiwen Li, Xian Wu, Jianjun Dong, Dan Zhang, Shuai Gao

**Affiliations:** 1 School of Business Administration, Liaoning Technical University, Huludao, Liaoning, China; 2 College of Safety Science and Engineering, Liaoning Technical University, Huludao, Liaoning, China; 3 Key Laboratory of Mine Thermodynamic Disasters and Control of Ministry of Education, Liaoning Technical University, Huludao, Liaoning, China; University of Alicante, SPAIN

## Abstract

Feature extraction of electrical load plays a vital role in providing a reliable basis and guidance for power companies. In this paper, we propose a novel clustering algorithm named the Density-based Matrix Transformation (DBMT) Clustering method to extract features (peaks, valleys and trends) of electrical load curves. The main objective of the algorithm is to reorder the data items until the data items belonging to the same cluster are organized together; that is, the adjacent matrix is rearranged to the type of block diagonal. This method adaptively determines the number of clusters and filters out noise without input global parameters. Moreover, for the specific characteristics of raw electrical load data, we propose a variant of Dynamic Time Warp (DTW) distance, dsDTW, which aligns the peaks, valleys and trends of load curves meanwhile dealing with missing values in different situations. After feeding the dsDTW adjacent matrix to DBMT, the results indicate that our proposal can accurately extract the feature of the load curves compared to different clustering methods.

## 1 Introduction

Electrical load clustering is an important tool for electrical data analysis. It plays a vital role in providing a reliable basis and accurate guidance for power companies [1]. For example, clustering on similar customers improves the accuracy of the prediction results and reduces the complexity of analytical modes. Moreover, depending on the extracted load curve’s feature from users, power companies can devise Demand-Responds strategies to reduce and shift peak demand and increase the stability of the grid [2]. Furthermore, understanding the load curve shapes of customer groups can also enable the devising of more efficient tariff structures, pricing and preferential schemes [3].

Due to the wide range of application scenarios of load curve clustering, different pattern recognition methodologies, such as k-means, self-organized maps, fuzzy k-means, and hierarchical methods, have been applied and shown to enhance our understanding of the electrical loads’ feature and facilitate the study of customers’ electricity behavior [4–6]. However, to design a more efficient clustering method for electrical load data, we must have a good command of the characteristics of the data collected from smart meters. By observing the raw data, we find two critical problems that need to be specified. First of all, the electrical load peaks and valleys are not always appearing in exact time but shifting in a short-range. This phenomenon may result from the delay of meters, and more importantly, fluctuation in users’ daily activities. Secondly, there are a non-negligible amount of missing values in the raw data.

**Fig 1:**
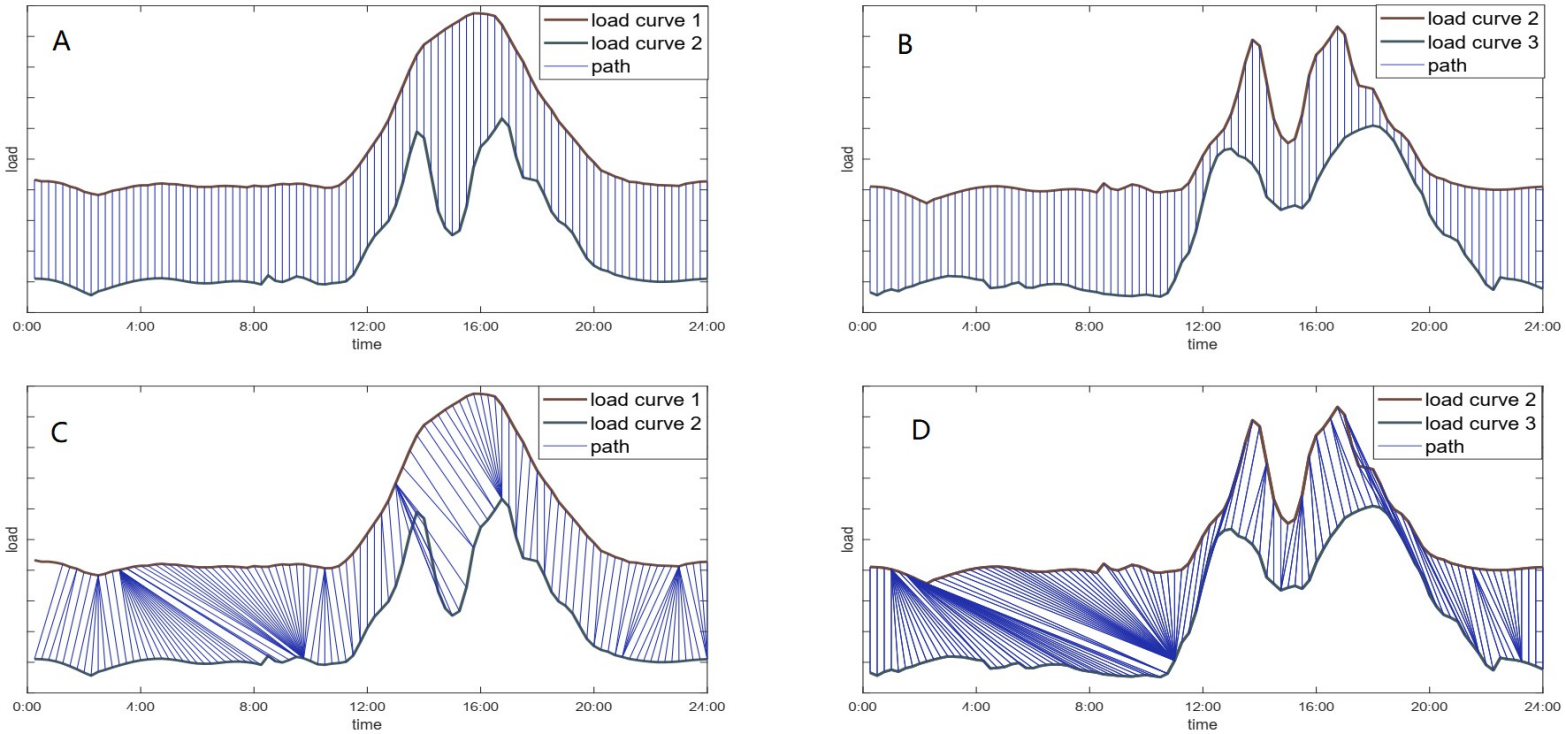
The comparison between Euclidean distance and DTW distance. (A) and (B) show the alignment rule of Euclidean. Ignoring the time shifts, the distance between the unimodal curve (load curve 1) and the unimodal curve (load curve 2), *d*^*EU*^(*curve*1, *curve*2) = 4.2742, is smaller than the distance between the unimodal curves (load curve 2 and load curve 3) *d*^*EU*^(*curve*2, *curve*3) = 4.8454. (C) and (D) on the contrary, show the alignment rules of DTW. Considering the time shifts, the distance between the unimodal curve (load curve 1) and the unimodal curve (curve 2), *d*^*DTW*^(*curve*1, *curve*2) = 0.0299, is larger than the distance between the unimodal curves (load curve 2 and load curve 3) *d*^*DTW*^(*curve*2, *curve*3) = 0.0099. Notes: in order to show the alignment clearly, we move down the lower curve for 2 units.

Many researchers focus on the first problem by applying new distance measurements. The distance between two items comes in many forms [7]. In Euclidean space, the most commonly-used and intuitive measurement of distance is Euclidean distance. It performs well when the attributes are unified. For efficiency only, squared Euclidean distance is used as a substitute. Given different backgrounds and applications, other distance measurements exist, such as Standardized Euclidean distance, Minkowski distance, Chebychev distance, Hamming distance, coefficient-type distances and graph-based distances [8]. However, due to the time-shifting, most of the mentioned measurements are no longer capable because they align the values at exactly the same time. On the contrary, Dynamic Time Warping (DTW) [9] is more flexible by considering the shape and trend of curve, aligning the peaks (valleys) to peaks (valleys). Consequently, the most important shape feature, number and location of peaks (valleys) are captured. In this way, load curves with the same number and location of peaks and valleys are more similar. We show the comparison of Euclidean distance and DTW distance in Fig 1. The Euclidean distance between the unimodal curve and the bimodal curve is smaller than the distance between two bimodal curves, while DTW is more reasonable to get a shorter distance between two bimodal curves compared with the distance between the unimodal curve and bimodal curve. This comparison shows the superiority of DTW distance to Euclidean distance in this application background. For the second missing values problem, the majority of existing clustering methods do not allow skipping time points in time series. The most commonly used solution is deleting all the data on the corresponding day, which causes a great information loss. According to the condition illustrated above, we proposed a variant of DTW, dsDTW, to align the trend of load curves meanwhile deal with missing values in different situations in Section 3.

Although DTW distance is a practical similarity measurement for time series, it’s not suitable to determine proper centers when embedded into many classical clustering methods. Without calculating centers, we propose a density-based method for electrical load curves based on the dsDTW adjacent matrix.

In our density-based clustering method, the key factor is to judge whether a data item belongs to an existing cluster, which is also vital in other clustering methods. For example, in the k-means algorithm, every data item is assigned to its nearest cluster, that is, the cluster with the shortest distance to it. However, using the distance between data item and cluster to determine the attribution of a data item is somewhat problematic. First, the position information (such as center or center of gravity) or representative values summarized from distances (such as mean value, maximum value, and minimum value) cannot reflect the real distribution of the data. Second, if the attribution of the data item is determined by comparison, an initial division is needed.

Our proposed clustering method can handle the mentioned problems. We study the distribution of distances between a selected data item and the rest of the data items in the data set. Information contained in the distribution is extracted and made full use. Moreover, we adopt a method similar to the well-known DBSCAN clustering algorithm. First, start with an initial data item, we extend the cluster until all the data items belonging to this cluster are found and labeled as one cluster. Then start a new cluster and repeat the previous step until all clusters are ascertained. The merits of the DBMT method are as follows. First, the parameters are data-driven from the local environment instead of global parameters or pre-determined thresholds. Consequently, it is still effective when the clusters are heterogeneous. Second, the number of clusters need not be given by users. Last, the proposed method operates on the adjacent matrix, so it does not need a definition of centers, which is a non-trivial case in cluster problems, especially when the DTW is used as similarity measurement.

The rest of the paper is organized as follows: In section 2, we introduce the DBMT clustering method in detail and show its effort on a simple spherical clustering data set. Section 3 shows how we construct the distance measurement, dsDTW, for load curves with missing values. Section 4 presents the case study on real-world electrical load curves. Finally, conclusions are drawn in section 5.

## 2 Algorithms

In this section, we describe the DBMT clustering algorithm in detail. At the beginning of this section, we present some fundamental conceptions. Second, we illustrate the central procedure of this algorithm, judgment on the 1st-neighbor (J1N), which determines splitting points between two clusters. Third, we describe the algorithm from an overall perspective.

### 2.1 Fundamental concepts

**Fig 2:**
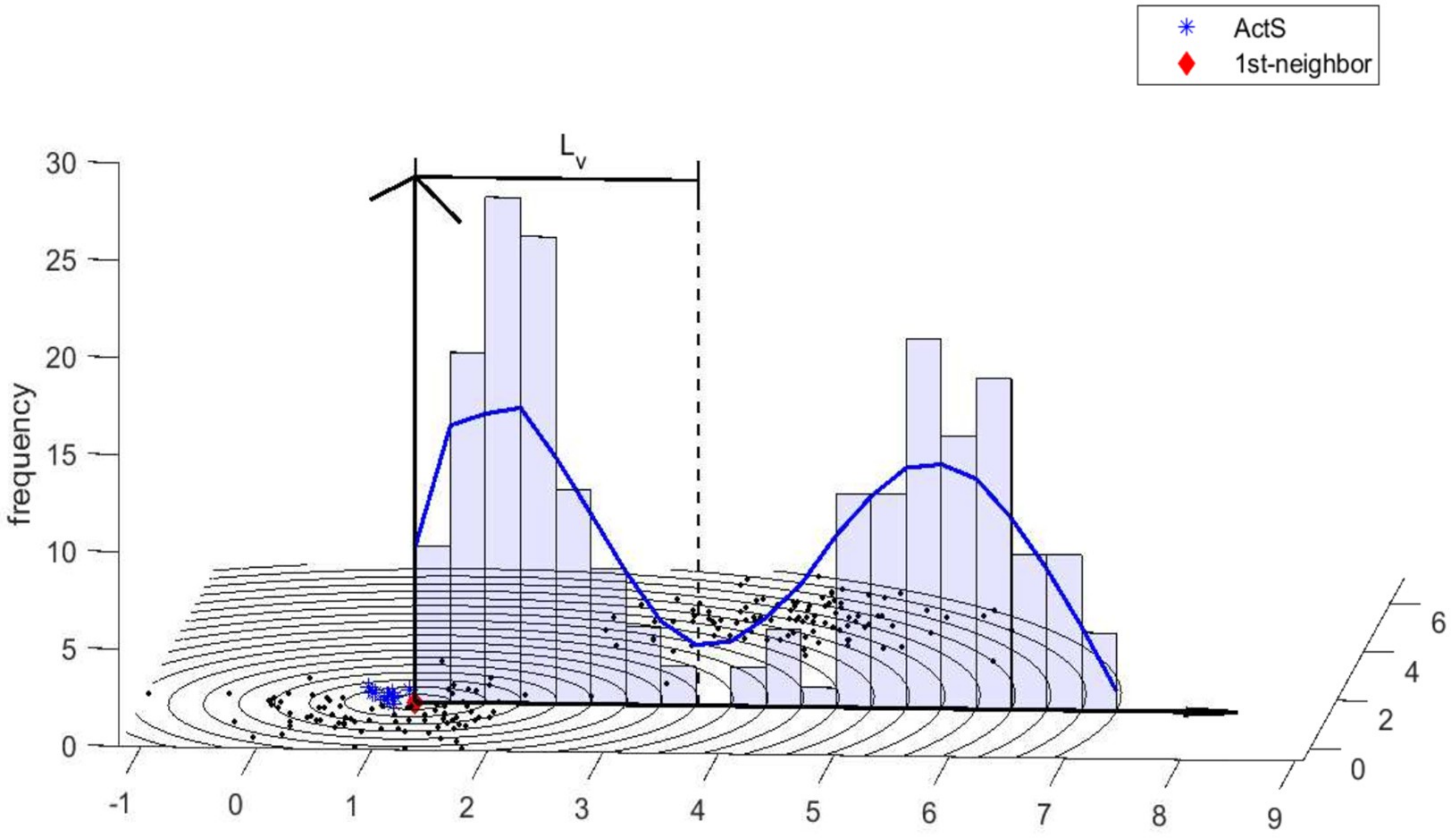
Presentation of active set and its 1st-neighbor. The distribution of distances between 1st-neighbor and all data items is presented; the histogram shows the frequency of distances; the corresponding blue line is smoothed frequency, acting as a approximated curve of probability density function (PDF).

**Conception 1**. ***[Distribution of distances]** D* = {*x*_1_, *x*_2_, …, *x*_*N*_} *is a data set, x*_*i*_ *is the i-th data item in D and N is the size of data set. The distances between x*_*i*_ *and all the data items are* {*d*(*x*_*i*_, *x*_1_), *d*(*x*_*i*_, *x*_2_), …, *d*(*x*_*i*_, *x*_*N*_)}; *these distances form a histogram* Fig 2. *Intuitively, we call it the distribution of distances and smooth it to a approximated curve of probability density function. The distribution of distances reflects how the data items scatter around x*_*i*_.

**Conception 2**. ***[Active Set]** We establish an active set, denoted as ActS*. *This active set is always a subset of one cluster. Without loss of generality, assuming* $ActS\in C_{i}=\left\{x_{1},x_{2},...,x_{N_{i}}\right\}$, *where C*_*i*_ *represents one of the clusters, N*_*i*_ *is the number of data items in C*_*i*_. *The data items in ActS are* $\left\{x_{1}^{ActS},x_{2}^{ActS},...,x_{N_{ActS}}^{ActS}\right\}$, *where N*_*ActS*_ *is the number of data items in ActS. The active subset is not static, but it begins as a single data item and is finished as a cluster. This process will be illustrated in Part 2.3*.

**Conception 3**. ***[1st-neighbor]** The concept of 1st-neighbor is given concerning the active set. Assuming that the unlabeled data items in the data set are* $\left\{x_{1}^{\prime },x_{2}^{\prime },...,x_{N_{unlabeled}}^{\prime }\right\}$, *where N*_*unlabeled*_ *is the total number of unlabeled items. We define the distance between* $x_{i}^{\prime }$ *and ActS as*

$$\begin{array}{c} d\left(x_{i}^{\prime },ActS\right)=\frac{1}{N_{ActS}}\Sigma_{j=1}^{N_{ActS}}d\left(x_{i}^{\prime },x_{ActS_{j}}\right) \end{array}$$ (1)

Simple but efficient, the 1st-neighbor of *ActS* is the data item whose average distance to all the members in *ActS* is minimum. (Note: From the viewpoint of statistics, the 1st-neighbor has the highest probability of belonging to the same cluster with *ActS*. Whereas we’d like to manage a practical engineering problem, we will not illustrate the statistical model here.) Denoted as *x*_1*n*_ (See Fig 2.) Analog to the hierarchical clustering method, we applied other distance measurements, such as minimum distance, maximum distance and single linkage. These alternative distance measurements are not as stable as average distance when noise exists, that is because the average distance shows the relative location between the *ActS* and its 1st-neighbor.

### 2.2 Judgment on the 1st-neighbor

To make a judgment on whether the active set *ActS* and its 1st-neighbor *x*_1*n*_ belong to one cluster, we establish the distribution of the distance between *x*_1*n*_ and the rest of the data items. In this distribution, the location of the first valley (*L*_*v*_) is a crucial data-driven threshold. It roughly represents the boundary between the cluster to which it belongs and the other clusters. So the criterion of judgment on whether the active set *ActS* and its 1st-neighbor *x*_1*n*_ belong to one cluster *C*_*k*_ is as follows, where *k* denotes the label of *C*_*k*_, if *ActS* ⊂ *C*_*k*_, then

$$\left\{ \begin{array}{c} x_{1n}\in C_{k},\;if\;d(x_{\text{1}n},ActS)\leq L_{v},\\ x_{1n}\notin C_{k},\;if\;d(x_{\text{1}n},ActS)>L_{v}. \end{array}\right.$$ (2)

If *x*_1*n*_ ∉ *C*_*k*_, it is noted as a splitting point. The corresponding algorithm is shown in Algorithm 1 J1N, where the *Adjc* represents the *N*-by-*N* adjacent matrix calculated by distance measurement (dsDTW).

Algorithm 1 J1N

**Require**: *x*_1*n*_, *Adjc*(*x*_1*n*_, :).

**Ensure**: 0/1

1: *freq* ← *hist*(*Adjc*(*x*_1*n*_, :))

2: *freq*_*smoothed*_ ← *Smooth*(*freq*)

3: *L*_*v*_ ← *first* *valley* *on* *freq*_*smoothed*_

4: **if** *L*_*v*_ < *mean*(*Adjc*(*x*_1*n*_, *S*_*ac*_)) **then**

5:  *return* 1

6: **else**

7:  *return* 0

8: **end if**

### 2.3 Main algorithm

In the first step, we establish an adjacent matrix with a distance measurement. The adjacent matrix should be established according to the characteristics of the application background. The dsDTW adjacent matrix is used in the load curve clustering problem due to the characteristic of dsDTW studied in the next section.

Next, we initialize the active set *ActS* to a single data item set. Then it gradually enlarges by absorbing its 1st-neighbor one by one if the J1N procedure returns 1. Once the active set and its 1st-neighbor no longer belong to one cluster, the active set is intuitively to be the cluster itself, *C*. That is, the 1st-neighbor of *ActS* is most likely to belong to the same cluster with *ActS* among all unlabeled data items. If *ActS* and its 1st-neighbor no longer belong to the same cluster, no other data item belongs to the same cluster with *ActS*. Then the clustering process for this current cluster is finished and the *x*_1*n*_ is noted as a splitting point, meanwhile, the active set is renewed to be a single point set with an unlabeled data item. Iteratively, the main algorithm is ended until all the data items are labeled. This is exactly the reason why the number of clusters is determined adaptively in the proposed method.

In practice, many tiny clusters are recognized at the end of the algorithm, rather than mix with each other or even impact the clustering result. They can be labeled as noise or abnormal load curves according to requirements and applications. In this way, our proposed method fulfills the request of detecting abnormal items.

**Fig 3:**
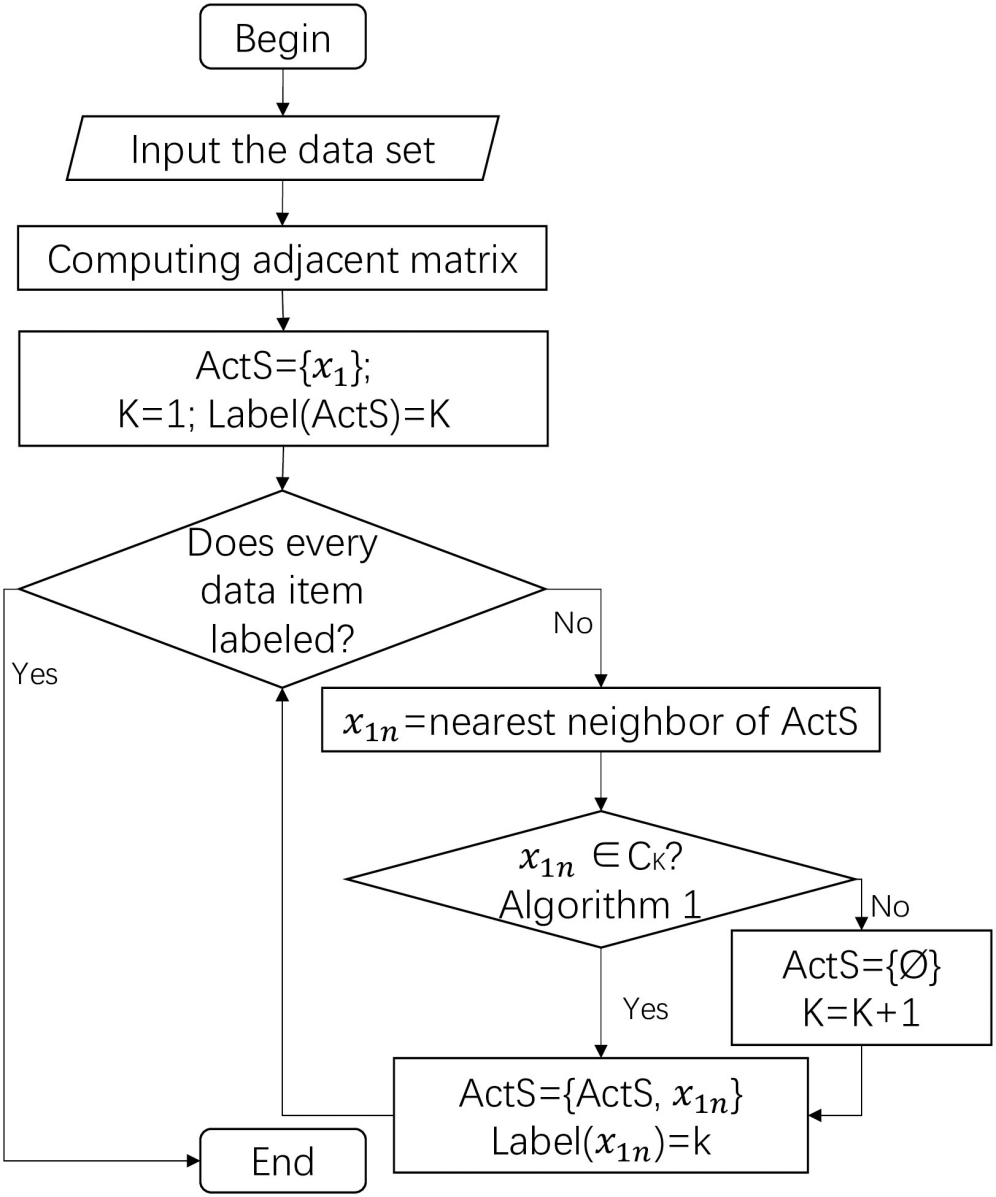
Work flow with 3 modules. (1) generater adjacent matrix; (2) judgement on 1st-neighbor and; (3) the integral loop layer.

Fig 3 presents the work flow of the DBMT method.

**Fig 4:**
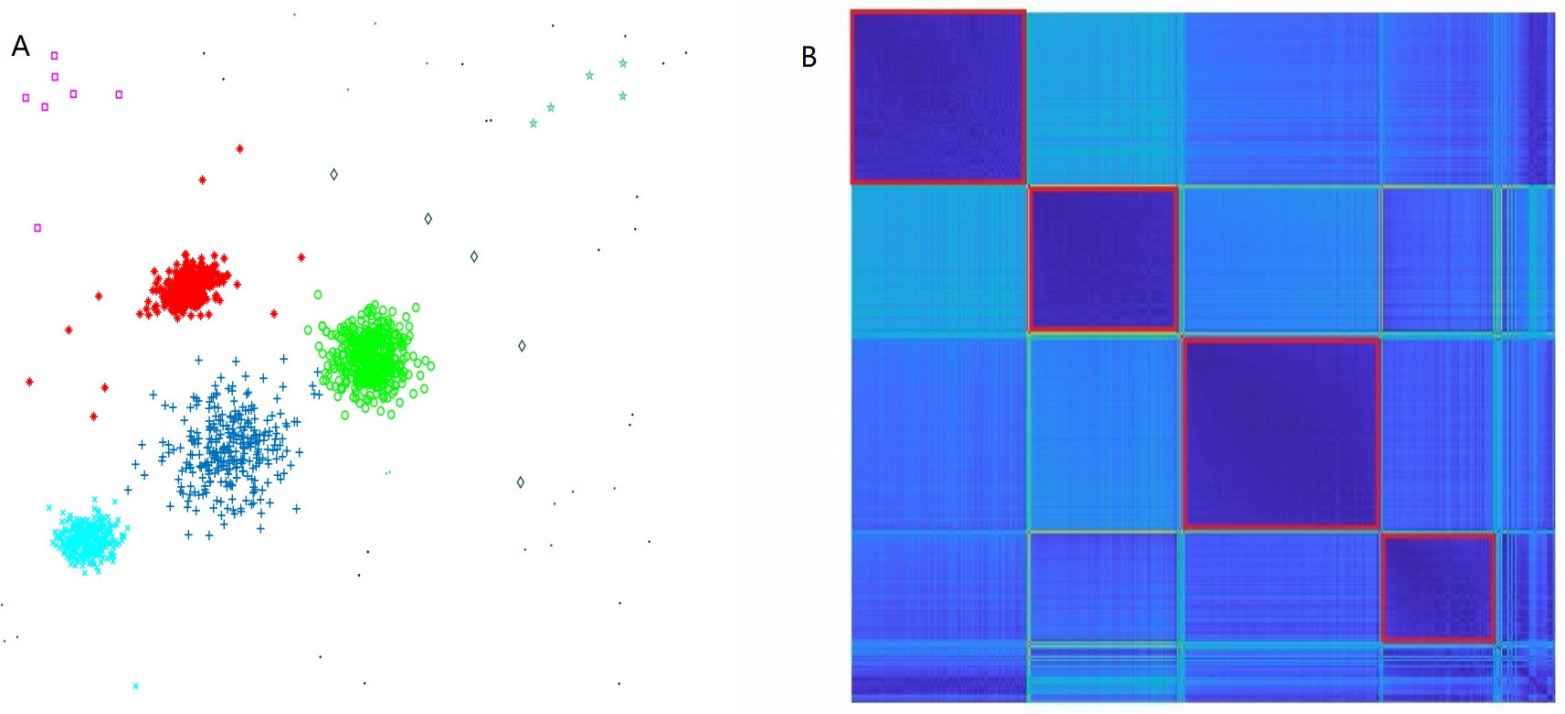
Heterogenous clusters with noise. (A) shows the clustering result with a scatter picture, in which the members belonging to the same clusters are shown in the same color and noise are labeled as tiny clusters; (B) shows the adjacent matrix after clustering, the blocks on the diagonal are separated clusters.

Moreover, we generate a data set composed of four clusters to verify that the DBMT algorithm can handle heterogeneous clusters and filter out noise. These four clusters are different in size and variance, and amid the noise, see Fig 4. (left), the clustering result is present in different colors and marks.

Before clustering, the adjacent matrix of the original data set is out-of-order. After iteratively applying J1N, the adjacent matrix is rearranged to block diagonal type; that is, the data items in one block are near to each other while the data items in different blocks are far from each other. Moreover, the number of apparent blocks is the number of clusters in the data set. The noise data items are labeled as tiny clusters with few members and then filtered out, see Fig 4(B).

### 2.4 Time complexity

The DBMT method is composed of two phases. In the first phase, the adjacent matrix is computed, which needs *O*(*N*^2^) time complexity. In the second phase, the main function is a circulatory process, in which one data item is labeled in one loop. In every loop, judgment is made, where the construction of distribution of distances needs a time complexity of *O*(*N*) at a time. For *N* data items, the whole time complexity of the second phase is *O*(*N*^2^). In conclusion, the total time complexity of the DBMT method is *O*(*N*^2^).

## 3 Missing value treatment

In this section, we discuss the treatment of missing values under different situations and propose a variant of DTW, named dsDTW.

DTW is one of the most crucial similarity measurement methods in time series data mining. Specific variants of DWT distance have been widely proposed to adapt to different fields, such a vector quantization DTW (VQ-DTW) for signature verification [10], statistical DTW for sign language recognition [11], and KNN-DTW for gene expression [12], name only a few. Analogously, in the load curve clustering, some variants of DTW distance are excellent substitutes for Euclidean distance in similarity measurement [13].

Computing DTW distance is dynamic programming, finding the minimum cumulative distance of each element on the align path (Fig 1). For time series *A* = (*a*_1_, *a*_2_, ⋯, *a*_*n*_) and *B* = (*b*_1_, *b*_2_, ⋯, *b*_*m*_), we need to find a path from (0, 0) to (*n*, *m*) in the *n* × *m* matrix, along which the sum of distance is minimized. DTW distance is defined as follows:

$$DTW\left(A,B\right)=\sqrt{dist(a_{n},b_{m})}$$

$$\begin{array}{c} dist\left(a_{i},b_{j}\right)={\left(a_{i}-b_{j}\right)}^{2}+min\{\begin{array}{r} dist(a_{i-1},\;b_{j})\\ dist(a_{i},\;b_{j-1})\\ dist(a_{i-1},\;b_{j-1}) \end{array} \end{array}$$ (3)

DTW matches the same feature, such as peaks, valleys and trends in different series, so it is more suitable to measure the dissimilarity between load curves for the peculiarity of load data. On the one hand, the records in the smart meters may mismatch in time for some environmental factors. On the other hand, customers’ daily activities are regular but not strict to exact times.

After applying DTW for time-shifting, the peaks are mapped to corresponding peaks, and so do the valleys. For example, people usually have breakfast around 8 am, 7:35 or 8:20, not fixing at an exact time. Consequently, the corresponding peaks on the load curve will not be strictly aligned. Reflected in the load curve, a time shift between peaks or valleys may exist. The similarity is more reasonable after aligning the same event on different days with DTW.

Nevertheless, this global matching strategy may not be suitable for all conditions. DTW explains all fluctuations in one series by warping the other series, leading to pathological results [14]. This means different events are aligned wrongly. This pitfall motivates research to propose many variants to mitigate this situation, such as, weighted DTW [15], Derivative Dynamic Time Warping (DDTW) [16] and Shape Contexts DTW [14]. However, the most common one is setting a window for warping, restricting the path to the diagonal region. Different structures of warping windows are proposed in [17] and dynamic time window strategy is presented in [18].

In our problem, two periods in different load curves are considered the same event only when a short time-shifting occurs. In this part, we utilize the Sakoe-Chuba Band (Band DTW) as the warping window to solve the pitfall of the pathological for the following reasons. On the one hand, in the load curve clustering problems, there will not be a long time-shifting for the same behavior of similar customers, such as having breakfast or supper, are constantly occurring around a fixed time, that is restricted in a *band*. On the other hand, using a band restriction is most explainable and intuitive, by which we treat all the time points equally. Meanwhile, a fixed band can preserve high accuracy with a lower running time. In this way, the same behaviors correspond to each other, and pathological results caused by over-warping are avoided. We will use *r* to present the length of the window in the following statement [19]. For the simpleness of the practical engineer problem, we set *r* to be 4 and illustrate it in the case study part.

Moreover, we realize the importance of handling missing values in load data when mining the raw data. For example, in the dataset we use in section 4, more than one-fifth of data items contain missing values. Consequently, handling data items with missing values is critical rather than discarding them. So we propose a refinement of Band DTW by considering the missing values under different situations (dsDTW).

**Fig 5:**
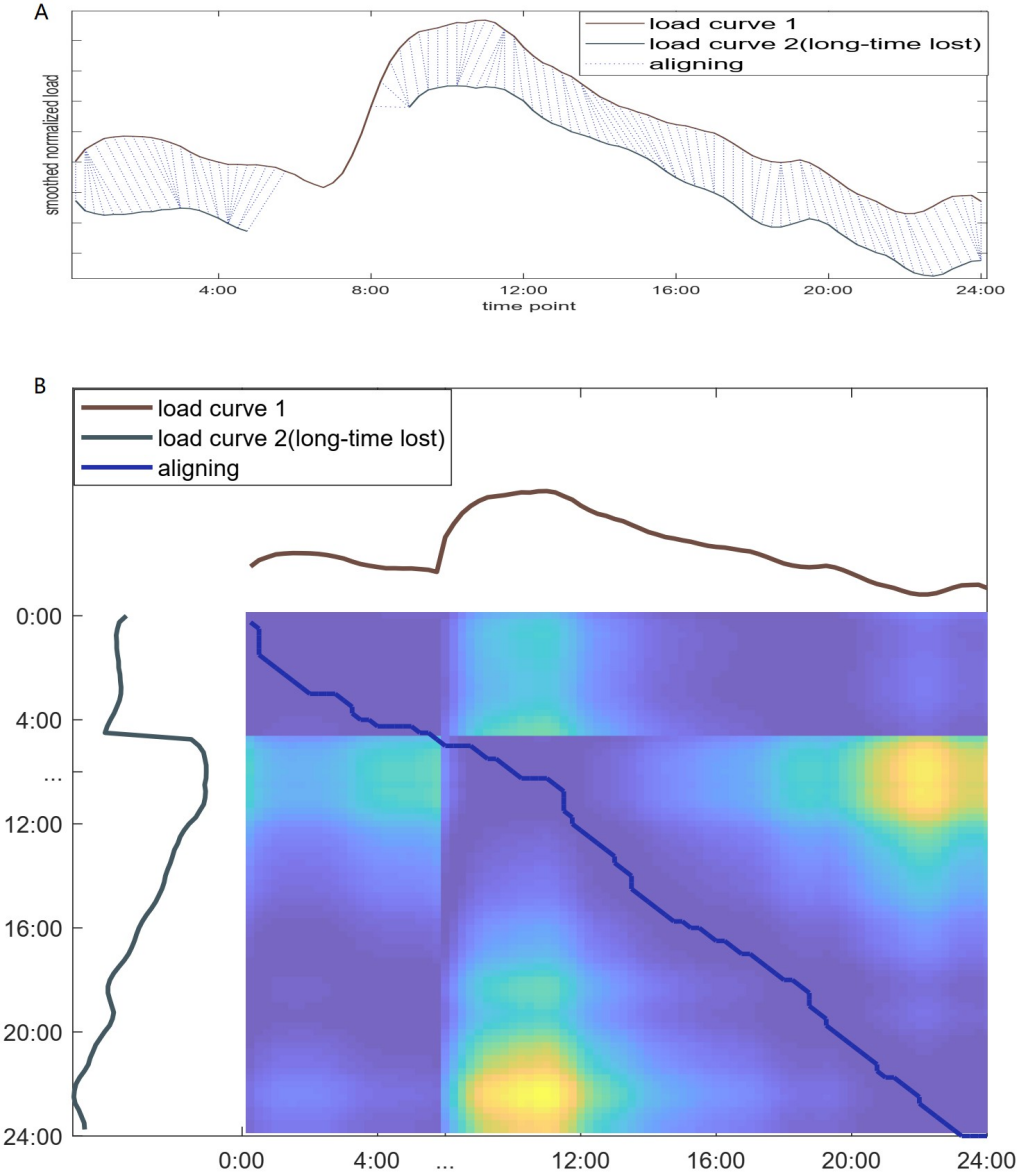
Long period missing values. (A) shows the long period missing values in load curve; (B) shows the heat map of dynamic warping and the new align path.

According to the volume of missing values, the missing values can be categorized into short-period and long-period. The short-period missing values can be interpolated according to their context in the curve. ARIMA is an ideal model for short-term forecasting. Two independent ARIMA models, a forward and a backward, are established using the records near the missing values. The average of the two estimated series is used to fill the missing values for robustness. In this way, the short-period missing values in load curves are filled accurately. Thus short-term missing values will not impact the overall feature of the load curve. While the long period of missing values can not be filled accurately, they should be treated differently because deleting the missing values directly leads to a disorder in the warping window in the band DTW. We present the proposed methodology in Fig 5. The upper subfigure shows two curves, with load curve 2 missing some values for a long period. In the first step, we reserve the sites of missing values and then add the band constraint on the path. Second, we establish a new matrix with renewed constraints by deleting the rows or columns corresponding to the missing values. Third, we calculate the DTW distance on the new matrix and constraints, see Fig 5 (lower). The matching result is shown in Fig 5 (upper). If a long section of values is missing in one curve, the corresponding part in the other curve is also invalid, which equals partitioning the calculation into two parts. Moreover, two details are to be illustrated in particular:

1. We set the threshold of missing length between short period and long period equaling to the warping window *r*; if the length of the missing segment exceeds *r*, the curve is divided into two parts. This setting is intuitive according to the motivation of the warping window for preventing over-warping.
2. A long path means more alignments being added, so we divided the sum of squares by the length of the path for fairness before square root calculation.

**Fig 6:**
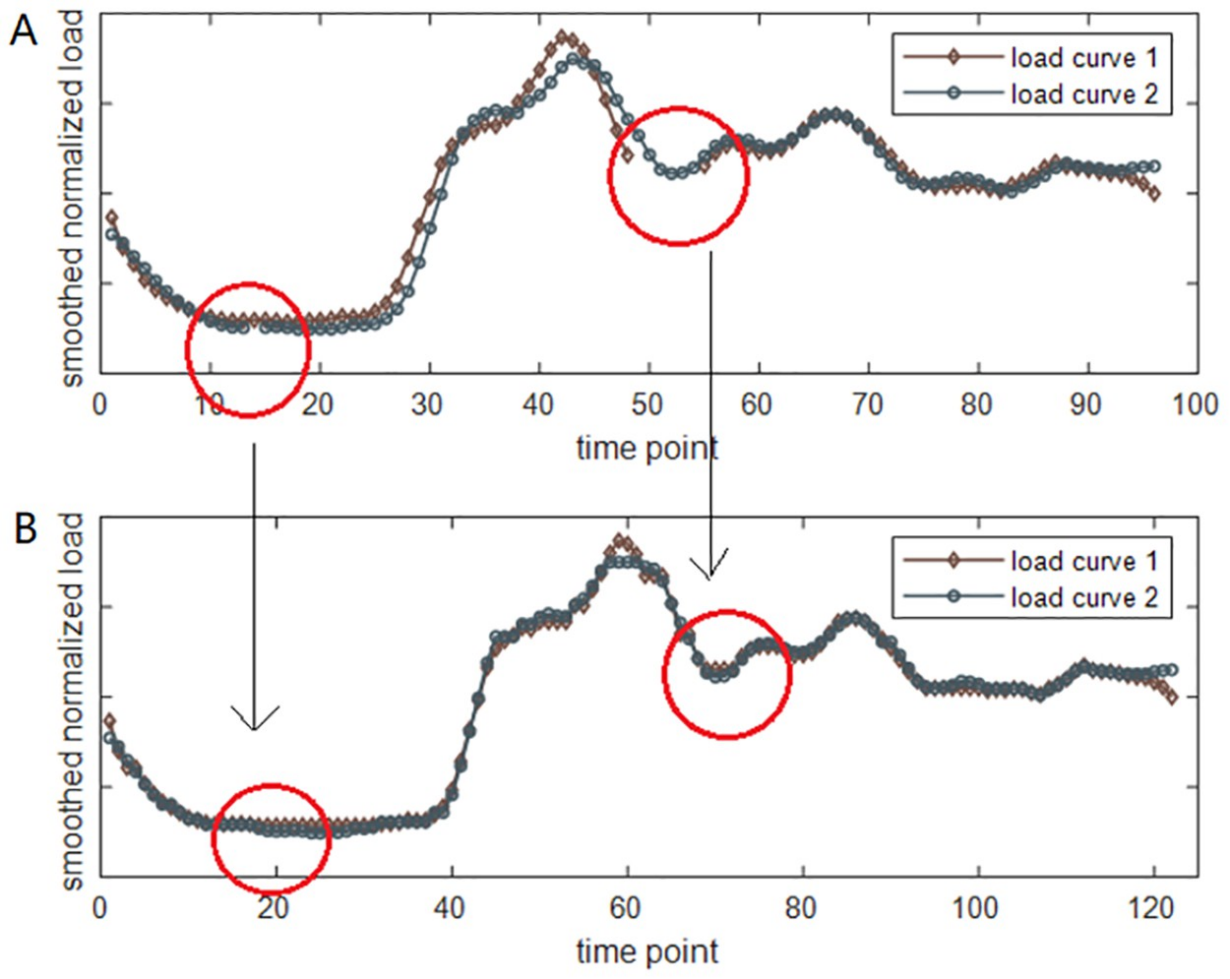
The applications of DTW on load curves with missing values. (A) The red cycles highlight the missing values in raw data. (B) The missing values are repaired by dsDTW.

To show the effect of dsDTW, we present a toy example, which contains both short-term and long-term missing values in Fig 6. The original data is shown in the upper figure; there are missing values in the red circles. The alignments generated by dsDTW are shown in the lower figure. The short-term missing values are repaired at the right time, and the long-term missing values have no impact on trend alignment.

As we mentioned above, the dsDTW is a reasonable distance measurement for load curves, which are fed to DBMT as the adjacent matrix.

## 4 Case study

In this section, we apply the proposed clustering algorithm and distance measurement dsDTW to real-world data to verify its performance. The experimental data is collected from smart meters in an industrial park for 15 days in 2018. There are 3261 data items, and their time interval is 15 minutes, so the regular length of each data item is 96. The types of electricity customers include urban residents customers, commercial customers, general industrial customers, large industrial customers, and non-industrial customers. Furthermore, these different types of electricity customers can be ulteriorly divided into users engaged in various industries. For example, large industrial and general industrial electricity include electronic components manufacturing, textile clothing manufacturing, and metal tools manufacturing. Therefore, the features of data are relatively complex. Moreover, it is undeniable that load curve clustering is a big challenge due to constant and unpredictable fluctuations. However, this condition is mitigated in our case study. On the one hand, extreme weather or equipment damage did not occur in our selected time period, which ensures the stableness of the load curve feature from a holistic perspective. On the other hand, The data is collected from one industry zone for continuous 15 days and each data item represents the daily load of one Distribution Station Area. Consequently, the behavior of users’ daily activities is stable and the data show strong periodicity. Thus, the data shows users’ normal daily routine and operating activities, which presents obvious aggregation characteristics.

Moreover, we normalized the data items to eliminate the impact of the magnitude. At the same time, we smoothed the daily load curves to reduce random fluctuations. After all the preparations, the dsDTW distances between the smoothed data items are calculated.

### 4.1 Discussion on parameters

**Fig 7:**
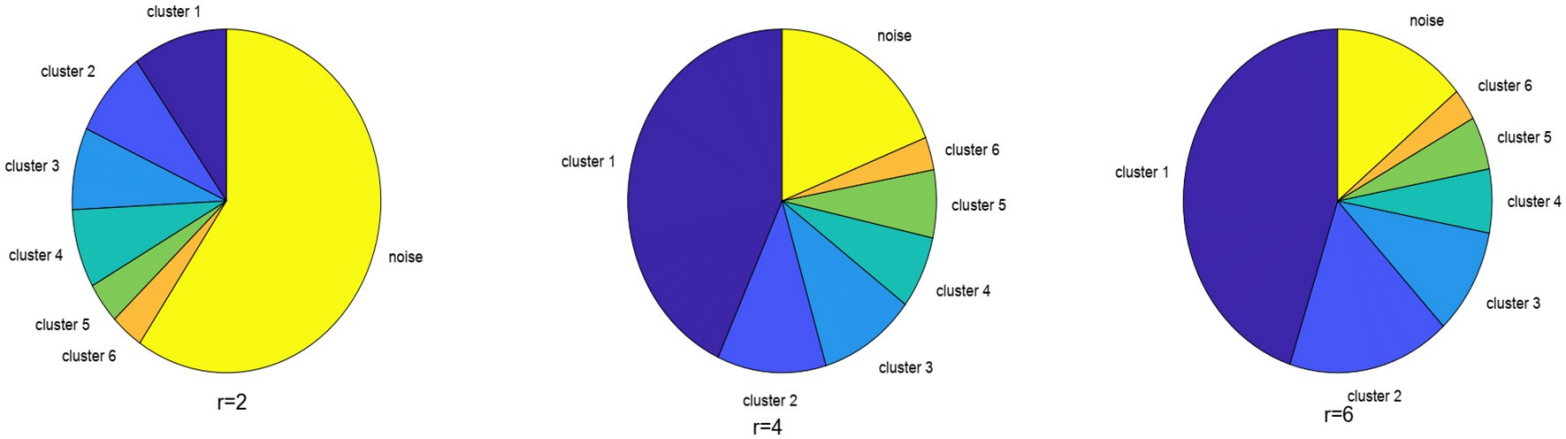
The proportions of clusters and noises under the different values of *r*. When *r* is set to be 2, most of the load curves are excluded from the six major clusters, that is, normal load curves are wrongly recognized as noise. When *r* is set to be 6, cluster 2 are composed of two types of different load curves, that is, the time tolerance is too high to divide clusters properly. *r* = 4 makes a trade-off between the mentioned situations.

**Fig 8:**
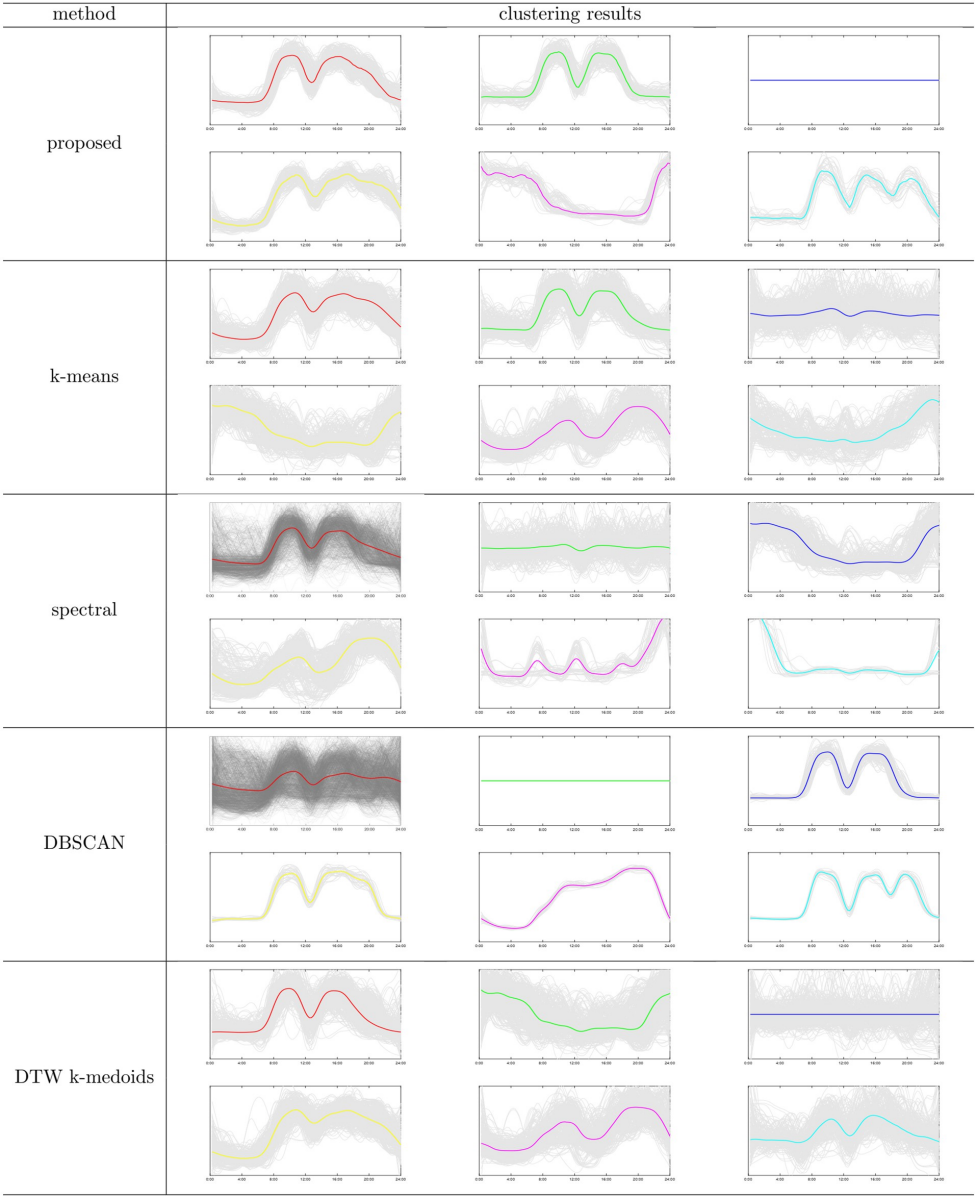
Clustering results of different clustering methods. Notes: The proposed method gets 6 major clusters and many tiny clusters. The tiny clusters are composed of a few members. In applications, these tiny clusters are recognized as noise. So we only present the 6 major clusters for conciseness.

The distance measurement, dsDTW, needs the warping window *r* given as a parameter. In this part, we set *r* to 2, 4, 6 to see its impact on the clustering result. The larger the *r*, the more tolerance to time-shifting and the less probability a curve is recognized as noise. As we can see in the pie charts in Fig 7, the noise ratio is decreased with the increase of *r*. In practical problems, we should make a trade-off, that is as much as load curves are assigned to corresponding clusters (low noise ratio) meanwhile different features are not mixed. According to Fig 7, the majority of load curves are labeled as tiny clusters or rather noises when *r* = 2. Meanwhile, two types of load curves with different features (the two clusters with three peaks in Fig 8 proposed) are mixed together when *r* = 6. Thus we set *r* to be 4, which is consistent with the advice of electrical engineering professionals. Moreover, *r* = 4 means a one-hour time-shifting. According to our life experience, the behavior fluctuation in users’ daily activities is on the hour scale.

### 4.2 Discussion on accuracy

The clustering results are shown in Fig 8. In addition to the proposed DBMT method, we also apply another three classic clustering methods, k-means, spectral clustering, DBSCAN, and another DTW-based clustering method noted as DTW k-medoids [13].

The DBMT gets six distinct clusters and many tiny clusters not shown in Fig 8. The first cluster accounts for 43.2% of the total data. It has a bimodal feature. These two peaks represent the working hours in the morning and afternoon, respectively, and the load keeps high after 7 am. The second cluster accounts for 11.4% percent of the total data. It is similar to the first cluster with two load peaks, while the main difference is the second peak formed at night time. The loads in the third cluster are zero all day, accounting for 10.12%. Querying to the industrial registration, they correspond to holidays and weekends of organizations, such as banks. The fourth cluster accounts for 6.82% of the whole data. It has three peaks but the peak at night is slightly lower than the peak in the afternoon, so it is not as obvious as the sixth cluster. The fifth cluster accounts for 6.38% of the total data. It shows the opposite of the first cluster and second cluster precisely; the load in the daytime is low and high at night. After a query, most load curves belong to large industries. Therefore, it verifies the effectiveness of the Time-of-Use pricing strategy. Large industries use enormous electricity, so they are sensitive to the electric price. Therefore, as a response to the Time-of-Use, they engage in productive activities at night when the electric price is low. The sixth cluster accounts for 3.1% of the total data with three distinct peaks compared with the fourth cluster. The rest of the load curves are labeled as noise with features different from the above six clusters.

Furthermore, we use the good result of our proposed method as pre-knowledge to determine the parameter in the k-means. The number of clusters in k-means is set to be 6. K-means algorithm potentially assumes that all the clusters are homogenous, so it performs poorly in such a complex real-world data set. The existence of noise even made the results worse. Similarly, we also set the number of clusters in the spectral clustering method to be 6; additionally, the *σ* in the kernel function is set to the best one detected by trial-and-error. However, the clustering result does not show distinct features between clusters. The parameters in DBSCAN are set according to the original paper [20], where *Minpts* = 4, and *eps* is set to the elbow point on the *Minpts*-distance plot. The first cluster includes the majority of the data, so the ability for feature extraction of DBSCAN in this data is weak. Lastly, the number of clusters in DTW k-medoids is set to be 6. The result shows that k-medoids tends to partition the curves into equal size, probably caused by local convergence.

### 4.3 Discussion on amount of data

Except for the DBMT method, all four other methods cannot deal with missing values. 2513 pieces of data items are obtained after filtering out the data items with missing values. So the size of data items in these algorithms is 2513. For the DBMT clustering method, there is one more thing that needs to be specified: a data item would also be eliminated if there is a half-day of value missing on one data item for the reason that the feature is not complete and the feature extraction for this data item is meaningless. So the number of data items handled by the DBMT method is 3086 after filtering out the data items with the number of missing values exceeding 48. We can see that around one-fifth of data items are wasted if the method can not handle the missing values. So the DBMT method makes full use of the original data, and it is more general than other methods because it is based on the adjacent matrix. We also believe this adjacent matrix-based algorithm can be applied to other fields.

## 5 Conclusion

In this paper, we proposed a density-based matrix transformation clustering algorithm operated on an adjacent matrix. For the particularity of power load curves, dsDTW is proposed to measure the distance between load curves with missing values, and then it is applied to calculate the adjacent matrix. The essential technique in DBMT is making a judgment on whether an active set and its 1st-neighbor *x*_1*n*_ belong to the same cluster. The distribution of the distances between *x*_1*n*_ and the rest data items is studied. We illustrate the reasonability of using the distribution of distances intuitively. Furthermore, the J1N process recognizes the splitting point, so there is no need to set the number of clusters as an input parameter. Meanwhile, the critical parameter *L*_*v*_ is driven out by data, so it is a suitable local parameter, which increases the algorithm’s flexibility over using a fixed global parameter. This is the reason why the DBMT is still valid in heterogeneous clusters. Last, we study real-world load data, which shows the validity of dsDTW and the proposed clustering method.
